# Supplementary material for: Impact of MgtC on the Fitness of Yersinia pseudotuberculosis
Source: Pathogens. 2023 Dec 8;12(12):1428. doi: 10.3390/pathogens12121428 (PMC10747817; doi:10.3390/pathogens12121428)
Supplement: Supplementary file 1 [file pathogens-12-01428-s001.zip › Additional file 1.pdf]

Table S1. Primers used in the present study.

Primer name	Sequence[a] (5' to 3')	Target gene[b]
mgtC-UF	cggcccgggtatatctttgagctgtgcatccccaa	Upstream fragment of *mgtC*
mgtC-UR	actcgtcaggccggagttaatgacatgattctctccatga	Upstream fragment of *mgtC*
mgtC-DF	tcatgtcattaactccggcctgacgagtcattaattaagcg	Downstream fragment of *mgtC*
mgtC-DR	cggggtacccgcataacgcttctcttttaattca	Downstream fragment of *mgtC*
YPTS_2313-UF	cggggtaccagattcccttttgcttgattca	Upstream fragment of *YPTS_2313*
YPTS_2313-UR	ctgtctccaactcaatgggggattcgttcctcagtgttctatt	Upstream fragment of *YPTS_2313*
YPTS_2313-DF	aatagaacactgaggaacgaatcccccattgagttggagacag	Downstream fragment of *YPTS_2313*
YPTS_2313-DR	cggcccgggcaacgcgacggcttggtcaata	Downstream fragment of *YPTS_2313*
YPTS_3071-UF	cggggtacctttccaaaactttagcgacttc	Upstream fragment of *YPTS_3071*
YPTS_3071-UR	acaaactctgaggatttgaaaggtcgcccccagaggttgtatca	Upstream fragment of *YPTS_3071*
YPTS_3071-DF	tgatacaacctctgggggcgacctttcaaatcctcagagtttgt	Downstream fragment of *YPTS_3071*
YPTS_3071-DF	cggcccgggtatgagatgccttgtctgtcga	Downstream fragment of *YPTS_3071*
mgtC-F	cggggatccctcgacggtaaatgtcgagggtatta	*mgtC* containing the promoter region
mgtC-R	cggggtaccactcgtcaggccggtaattcagatg	*mgtC* containing the promoter region

YPTS_2313-F	cggg<u>aattc</u>attaattttgagatccactgtct	*YPTS_2313* containing the promoter region
YPTS_2313-R	cgg<u>aagctt</u>ctatttagccgctggtgctac	*YPTS_2313* containing the promoter region
YPTS_3071-F	cggg<u>aattc</u>atccgctattcagggataaggc	*YPTS_3071* containing the promoter region
YPTS_3071-R	cgg<u>aagctt</u>ttatgctgcgggtgccgctact	*YPTS_3071* containing the promoter region
RT-*rpoD*-F	cgatgtcacccgtgaacgta	*rpoD*
RT-*rpoD*-R	aatcgtcgaggaagctacgc	*rpoD*
RT-*mgtC*-F	taaatctatcgcctgctgttga	*mgtC*
RT-*mgtC*-R	gctttgtggtctgggtttgtac	*mgtC*
RT-*YPTS_2313*-F	taacgcatcagctaaaaccatg	*YPTS_2313*
RT-*YPTS_2313*-R	tggctatgtgccgctttatggt	*YPTS_2313*
RT-*YPTS_0225*-F	catggttgagcctggcggacat	*YPTS_0225*
RT-*YPTS_0225*-R	ccctgatcctgtaaggcactcg	*YPTS_0225*
RT-*YPTS_0224*-F	tgatggcactgtcgatcctttc	*YPTS_0224*
RT-*YPTS_0224*-R	ctgccgcaacaaaccgataact	*YPTS_0224*
RT-*YPTS_0091*-F	atggcggtaatgactgtttcaa	*YPTS_0091*
RT-*YPTS_0091*-R	tccaactggcaggtatcttctc	*YPTS_0091*

RT-*YPTS_3071*-F	ctttagtttgagcagcaacagt	*YPTS_3071*
RT-*YPTS_3071*-R	ccaccctgggtctatcttcagt	*YPTS_3071*
RT-*YPTS_0907*-F	ggaagacatggtattaagcgagat	*YPTS_0907*
RT-*YPTS_0907*-R	ccagattgccagttgccagata	*YPTS_0907*
RT-*YPTS_1642*-F	attggcgttaccatcgaagtct	*YPTS_1642*
RT-*YPTS_1642*-R	cactgctattcagggcgagatt	*YPTS_1642*
RT-*YPTS_3843*-F	caactcagcggcggtgataggt	*YPTS_3843*
RT-*YPTS_3843*-R	tggcagatacggtgatggacgt	*YPTS_3843*
RT-*YPTS_2011*-F	gccgcacgctgtttacggtctc	*YPTS_2011*
RT-*YPTS_2011*-R	gtgttccgctgaagcaaggtca	*YPTS_2011*
RT-*YPTS_3842*-F	tcgcaatctgaggtcaataaatc	*YPTS_3842*
RT-*YPTS_3842*-R	tatgggcggtaaggtactggtg	*YPTS_3842*

a Underlining indicates restriction endonuclease recognition sequences.
b Y. pseudotuberculosis locus tags listed are for genes in Y. pseudotuberculosis PB1+.
