# Supplementary material for: Impact of MgtC on the Fitness of Yersinia pseudotuberculosis
Source: Pathogens. 2023 Dec 8;12(12):1428. doi: 10.3390/pathogens12121428 (PMC10747817; doi:10.3390/pathogens12121428)
Supplement: Supplementary file 1 [file pathogens-12-01428-s001.zip › Additional file 2.pdf]

Table S2 List of DEGs between PB1+ and Δ*mgtC* mutant within macrophages

Gene number	Gene name	Fold change Δ*mgtC*/ PB1+	*p*-value
YPTS_0023	nitrogen regulation protein NR(I)	2.147149827	5.81E-07
YPTS_0024	nitrogen regulation protein NR(II)	2.525869672	0.003252097
YPTS_0025	glutamate--ammonia ligase	2.209754743	2.43E-09
YPTS_0078	ABC transporter substrate-binding protein	2.394514719	4.50E-11
YPTS_0090	glycerol kinase GlpK	0.323951542	1.78E-26
YPTS_0091	aquaporin family protein	0.219090038	1.55E-28
YPTS_0110	methylenetetrahydrofolate reductase	2.711845614	1.52E-08
YPTS_0183	UDP-N-acetyl-D-mannosamine dehydrogenase	0.413035902	4.77E-18
YPTS_0184	dTDP-glucose 4,6-dehydratase	0.465528239	1.20E-15
YPTS_RS01165	hypothetical protein	0.475835831	0.000713797
YPTS_0224	anaerobic glycerol-3-phosphate dehydrogenase subunit A	0.236100615	1.77E-33
YPTS_0225	glycerol-3-phosphate dehydrogenase subunit GlpB	0.260504109	1.03E-31
YPTS_0226	anaerobic glycerol-3-phosphate dehydrogenase subunit C	0.378057445	3.30E-18

YPTS_0241	branched-chain amino acid ABC transporter substrate-binding protein	2.201098639	1.31E-06
YPTS_0263	HTH-type transcriptional regulator MetR	2.147763459	6.09E-05
YPTS_0264	5-methyltetrahydropteroyltriglutamate--homocysteine S-methyltransferase	2.074917763	1.40E-09
YPTS_0282	acetyl-CoA C-acyltransferase FadA	2.466842652	2.41E-11
YPTS_0283	fatty acid oxidation complex subunit alpha FadB	2.058822243	2.20E-10
YPTS_0293	GNAT family N-acetyltransferase	2.294317315	2.29E-09
YPTS_0304	DNA-directed RNA polymerase subunit beta	0.455546385	2.88E-09
YPTS_0305	DNA-directed RNA polymerase subunit beta	0.347607115	1.82E-14
YPTS_0439	fumarate reductase (quinol) flavoprotein subunit	0.402613898	0.018157336
YPTS_0550	anaerobic ribonucleoside-triphosphate reductase	0.390709249	7.93E-09
YPTS_0571	4S)-4-hydroxy-5-phosphonooxypentane-2,3-dione isomerase	2.641278509	2.59E-11
YPTS_0572	3-hydroxy-5-phosphonooxypentane-2,4-dione thiolase	2.92107963	7.53E-12
YPTS_0573	autoinducer 2 ABC transporter substrate-binding protein LsrB	2.932189351	6.44E-19
YPTS_0574	autoinducer 2 ABC transporter permease LsrD	2.501774393	2.17E-10

YPTS_0575	autoinducer 2 ABC transporter permease LsrC	2.802127145	5.72E-15
YPTS_0576	autoinducer 2 ABC transporter ATP-binding protein LsrA	2.502428804	1.19E-13
YPTS_0577	transcriptional regulator LsrR	2.034727834	5.38E-09
YPTS_0578	autoinducer-2 kinase	2.590941396	1.82E-14
YPTS_0696	3-isopropylmalate dehydratase large subunit	0.485091038	4.90E-07
YPTS_0740	amino acid permease	2.459612297	2.07E-10
YPTS_0824	c-type cytochrome	0.458428333	2.26E-08
YPTS_0845	cytochrome b562	2.722238154	7.88E-09
YPTS_0846	catalase/peroxidase HPI	3.164272904	2.96E-13
YPTS_0907	transporter substrate-binding domain-containing protein	6.494780965	2.54E-07
YPTS_0908	amino acid ABC transporter permease	4.69526671	1.82E-05
YPTS_0909	amino acid ABC transporter permease	3.602257625	1.38E-07
YPTS_0919	S-methyl-5-thioribose kinase	2.240961091	3.09E-06
YPTS_1005	ComEA family DNA-binding protein	2.381736691	4.12E-12
YPTS_1014	P-II family nitrogen regulator	3.52346994	1.05E-05
YPTS_1015	ammonium transporter AmtB	3.836423497	6.52E-05

YPTS_1023	50S ribosomal protein L36	0.43011056	2.29E-10
YPTS_1132	ABC transporter permease	2.172144933	0.000340324
YPTS_1133	Gfo/Idh/MocA family oxidoreductase	2.075483552	5.54E-05
YPTS_1164	amino acid ABC transporter permease	2.391539086	2.07E-11
YPTS_1165	amino acid ABC transporter substrate-binding protein	2.869069076	2.59E-15
YPTS_RS06050	hypothetical protein	2.138355484	0.000218254
YPTS_1265	ABC transporter ATP-binding protein	3.86502002	2.73E-06
YPTS_1283	zinc resistance sensor/chaperone ZraP	2.174156779	1.50E-05
YPTS_1310	GlsB/YeaQ/YmgE family stress response membrane protein	2.427430937	4.86E-10
YPTS_1316	urea ABC transporter substrate-binding protein	4.54845303	2.12E-05
YPTS_1317	urea ABC transporter permease subunit UrtB	4.489934827	0.000982073
YPTS_1318	urea ABC transporter permease subunit UrtC	2.278188716	0.043567279
YPTS_1319	urea ABC transporter ATP-binding protein UrtD	2.503637213	0.033076257
YPTS_1320	urea ABC transporter ATP-binding subunit UrtE	2.769848622	0.004632756
YPTS_1412	C-terminal binding protein	2.045768007	0.000148772
YPTS_1413	fumarylacetoacetate hydrolase family protein	2.666662892	1.56E-09

YPTS_1415	aldehyde dehydrogenase family protein	3.082736094	1.64E-13
YPTS_1416	xylulokinase	2.593430791	1.69E-06
YPTS_1444	isopenicillin N synthase family oxygenase	2.315114244	2.00E-10
YPTS_1445	ABC transporter substrate-binding protein	2.732107005	4.86E-10
YPTS_1446	methionine ABC transporter ATP-binding protein	2.764805641	7.66E-10
YPTS_1447	ABC transporter permease	2.301040434	2.67E-08
YPTS_1492	cold shock-like protein CspD	2.429709503	2.76E-13
YPTS_1642	sugar ABC transporter substrate-binding protein	6.125790508	2.34E-05
YPTS_1685	MFS transporter	4.673309228	2.73E-06
YPTS_1686	thiol-disulfide oxidoreductase DCC family protein	2.080341145	1.84E-05
YPTS_1688	SDR family oxidoreductase	2.878130252	2.94E-13
YPTS_1689	fumarylacetoacetate hydrolase family protein	3.942438216	4.09E-19
YPTS_1690	L-fuconate dehydratase	3.296246497	1.41E-15
YPTS_1695	MFS transporter	2.74091403	2.77E-09
YPTS_1696	polysaccharide deacetylase	3.648973491	9.93E-15
YPTS_1710	yersiniabactin biosynthesis thioesterase YbtT	0.483503591	0.003506162

YPTS_1814	autoinducer 2 ABC transporter substrate-binding protein	2.155565731	2.21E-06
YPTS_1855	hypothetical protein	0.487364092	1.36E-05
YPTS_1961	Gfo/Idh/MocA family oxidoreductase	2.891991815	9.07E-08
YPTS_2005	phosphate starvation-inducible protein PhoH	2.434476246	6.91E-15
YPTS_2011	aspartate aminotransferase family protein	5.083721222	3.46E-06
YPTS_2012	arginine N-succinyltransferase	4.080613877	9.35E-05
YPTS_2013	succinylglutamate-semialdehyde dehydrogenase	3.268383034	0.000223275
YPTS_2014	N-succinylarginine dihydrolase	3.181079907	4.57E-12
YPTS_2015	succinylglutamate desuccinylase	2.937426506	8.02E-10
YPTS_2021	imidazolonepropionase	0.45259316	7.70E-07
YPTS_2195	BON domain-containing protein	2.401015691	1.38E-11
YPTS_2259	MFS transporter	0.391870666	2.61E-17
YPTS_2313	acid-shock protein	0.269330723	5.84E-21
YPTS_2356	YIP1 family protein	2.459117501	1.82E-14
YPTS_2490	MgtC family protein	0.000000953	8.20E-99
YPTS_2643	glutamine ABC transporter substrate-binding protein GlnH	2.243024614	5.38E-09

YPTS_2666	gluconate 5-dehydrogenase	0.484383816	6.54E-08
YPTS_2670	NADH-quinone oxidoreductase subunit NuoN	0.464977734	3.12E-12
YPTS_2695	PTS sugar transporter subunit IIB	0.431193795	3.13E-14
YPTS_2701	histidine ABC transporter ATP-binding protein HisP	2.754590379	0.002051971
YPTS_2702	ABC transporter permease subunit	2.977761796	0.003006144
YPTS_2703	ABC transporter permease subunit	3.535165988	0.000426441
YPTS_2704	histidine ABC transporter substrate-binding protein HisJ	3.634787612	6.52E-06
YPTS_2736	long-chain fatty acid transporter FadL	2.602374118	9.73E-13
YPTS_2831	cysteine synthase CysM	2.299971516	3.62E-08
YPTS_2832	sulfate/thiosulfate ABC transporter ATP-binding protein CysA	2.688614703	2.47E-12
YPTS_2833	sulfate/thiosulfate ABC transporter permease CysW	2.215453542	1.38E-08
YPTS_2834	sulfate/thiosulfate ABC transporter permease CysT	2.296583549	9.82E-10
YPTS_2876	ABC transporter substrate-binding protein	2.010223102	1.56E-09
YPTS_2925	DUF1508 domain-containing protein	2.100229635	8.94E-12
YPTS_3071	acid shock protein	0.169892076	3.40E-08
YPTS_3294	lysine--tRNA ligase	0.45192401	3.64E-13

YPTS_3310	lipocalin-like domain-containing protein	2.407340506	6.10E-10
YPTS_3792	aliphatic sulfonates ABC transporter ATP-binding protein	2.410116634	2.63E-07
YPTS_3794	FMNH2-dependent alkanesulfonate monooxygenase	2.678473672	0.003107434
YPTS_3840	glyoxylate bypass operon transcriptional repressor IclR	2.50468052	4.95E-09
YPTS_3841	bifunctional isocitrate dehydrogenase kinase/phosphatase	3.290116342	8.71E-13
YPTS_3842	isocitrate lyase	5.081315499	1.19E-07
YPTS_3843	malate synthase A	5.255803787	4.88E-09
YPTS_3892	bacterioferritin	2.099088125	8.15E-09
YPTS_3911	taurine dioxygenase	2.767316039	1.76E-11
YPTS_3912	taurine ABC transporter permease TauC	2.360295242	7.66E-09
YPTS_3979	glycerol-3-phosphate dehydrogenase	0.39641426	1.64E-13
YPTS_4021	DDE-type integrase/transposase/recombinase	2.097233419	9.15E-09
YPTS_4025	universal stress protein UspB	2.755884608	5.64E-10
YPTS_4058	ABC transporter substrate-binding protein	2.846840716	1.33E-15
YPTS_4063	uptakes hexose phophates and regulates uhpT	0.475293685	3.29E-08
YPTS_4184	glutamine--fructose-6-phosphate transaminase	0.498768698	2.75E-11

YPTS_4192	transporter substrate-binding domain-containing protein	2.290503493	1.12E-11
YPTS_4193	amino acid ABC transporter permease	2.253607139	1.52E-08
YPTS_RS21675	AAA family ATPase	2.682206191	0.001944559
YPTS_4183	hypothetical protein	0.490194232	0.000288531
