# Supplementary material for: Impact of MgtC on the Fitness of Yersinia pseudotuberculosis
Source: Pathogens. 2023 Dec 8;12(12):1428. doi: 10.3390/pathogens12121428 (PMC10747817; doi:10.3390/pathogens12121428)
Supplement: Supplementary file 1 [file pathogens-12-01428-s001.zip › Additional file 3.pdf]

S1

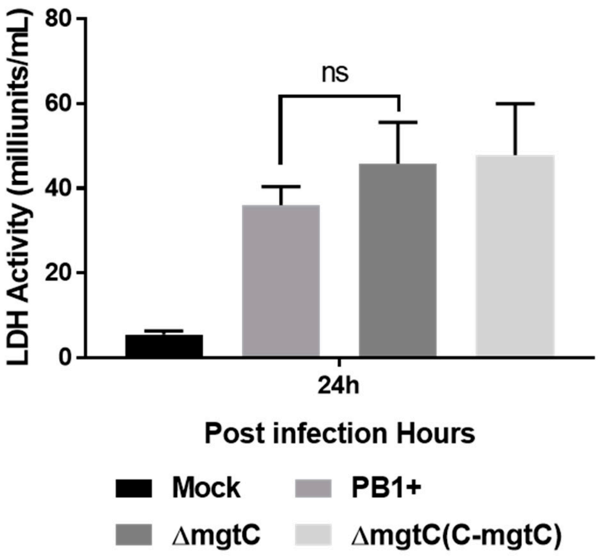

Figure S1. LDH detection. RAW264.7 cells were infected with WT PB1+, Δ*mgtC* and Δ*mgtC*(C-*mgtC*) at a MOI of 20. The supernatants were collected at 24 h p.i., and LDH release was detected using the LDH Activity Assay Kit. The supernatants of uninfected RAW264.7 cells were used as negative controls (medium). ns, no significant.The experiment was performed twice and pooled together.
