# Supplementary material for: Impact of MgtC on the Fitness of Yersinia pseudotuberculosis
Source: Pathogens. 2023 Dec 8;12(12):1428. doi: 10.3390/pathogens12121428 (PMC10747817; doi:10.3390/pathogens12121428)
Supplement: Supplementary file 1 [file pathogens-12-01428-s001.zip › Additional file 4.pdf]

A

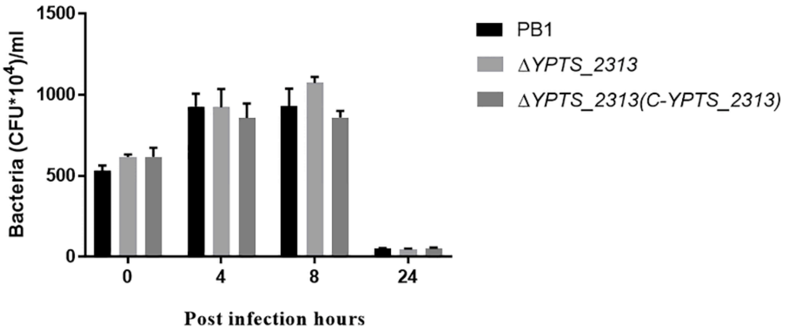

B

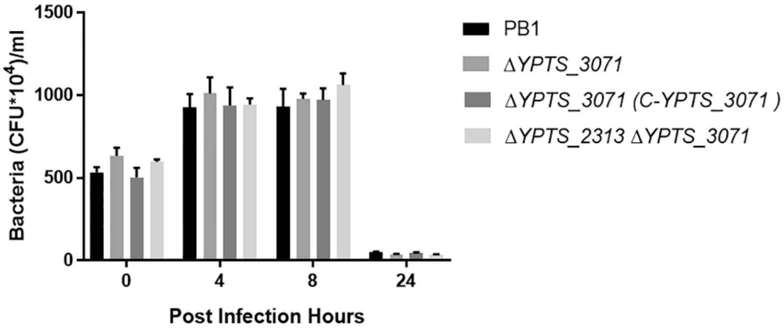

Figure S2. Intracellular survival within RAW 264.7 macrophages. RAW264.7 cells were infected with different *Y. pseudotuberculosis* strains at a MOI of 20. The infected cells were incubated with 0.2% (v/v) Triton X-100 at 0 h, 4 h, 8 h and 24 h p.i.. Then, intracellular CFUs of bacteria were enumerated. (A) RAW264.7 cells were infected with WT PB1+, Δ*YPTS_2313*, and Δ*YPTS_2313*(C-*YPTS_2313*) (B) RAW264.7 cells were infected with WT PB1+, Δ*YPTS_3071*, Δ*YPTS_2313*Δ*YPTS_3071* and Δ*YPTS_3071*(C-*YPTS_3071*). The experiment was performed twice and pooled together.
